# How do patient feedback systems work in low-income and middle-income countries? Insights from a realist evaluation in Bangladesh

**DOI:** 10.1136/bmjgh-2020-004357

**Published:** 2021-02-10

**Authors:** Tolib Mirzoev, Sumit Kane, Zunayed Al Azdi, Bassey Ebenso, Ayesha Afroz Chowdhury, Rumana Huque

**Affiliations:** 1 Nuffield Centre for International Health and Development, University of Leeds, Leeds, UK; 2 Nossal Institute for Global Health Melbourne School of Population and Global Health, The University of Melbourne, Melbourne, Victoria, Australia; 3 Research and Development, ARK Foundation, Dhaka, Bangladesh; 4 Department of Community Medicine, Sir Salimullah Medical College, Dhaka, Bangladesh; 5 Department of Economics, University of Dhaka, Dhaka, Bangladesh

**Keywords:** health systems evaluation, health policy, health services research, health systems

## Abstract

**Background:**

Well-functioning patient feedback systems can contribute to improved quality of healthcare and systems accountability. We used realist evaluation to examine patient feedback systems at health facilities in Bangladesh, informed by theories of citizenship and principal–agent relationships.

**Methods:**

We collected and analysed data in two stages, using: document review; secondary analysis of data from publicly available web-portals; in-depth interviews with patients, health workers and managers; non-participant observations of feedback environments; and stakeholder workshops. Stage 1 focused on identifying and articulating the initial programme theory (PT) of patient feedback systems. In stage 2, we iteratively tested and refined this initial theory, through analysing data and grounding emerging findings within substantive theories and empirical literature, to arrive at a refined PT.

**Results:**

Multiple patient feedback systems operate in Bangladesh, essentially comprising stages of collection, analysis and actions on feedback. Key contextual enablers include political commitment to accountability, whereas key constraints include limited patient awareness of feedback channels, lack of guidelines and documented processes, local political dynamics and priorities, institutional hierarchies and accountability relationships. Findings highlight that relational trust may be important for many people to exercise citizenship and providing feedback, and that appropriate policy and regulatory frameworks with clear lines of accountability are critical for ensuring effective patient feedback management within frontline healthcare facilities.

**Conclusion:**

Theories of citizenship and principal–agent relationships can help understand how feedback systems work through spotlighting the citizenship identity and agency, shared or competing interests, and information asymmetries. We extend the understanding of these theories by highlighting how patients, health workers and managers act as both principals and agents, and how information asymmetry and possible agency loss can be addressed. We highlight the importance of awareness raising and non-threatening environment to provide feedback, adequate support to staff to document and analyse feedback and timely actions on the information.

**Key questions**

**What is already known?**

- Well-functioning patient feedback systems can contribute to improved quality of healthcare and ultimately make health systems more accountable and responsive to local needs.
- Most published evidence comes from high-income countries and hospital settings with less research from lower-income countries and frontline health facilities, and this study used realist evaluation to examine patient feedback systems at frontline health facilities in Bangladesh.

**What are the new findings?**

- Multiple patient feedback systems operate in Bangladesh in the context of political commitment to accountability and responsiveness, but are constrained by limited patient awareness of feedback channels, lack of guidelines and documented processes, local political dynamics and priorities, institutional hierarchies and accountability relationships.
- Relational trust can be important for people to exercise their citizenship in providing feedback, and appropriate policy and regulatory frameworks with clear lines of accountability are critical for ensuring effective patient feedback management.

**What do the new findings imply?**

- Theories of citizenship and principal–agent relationships can help understand how feedback systems work through spotlighting the citizenship identity and agency, shared or competing interests, and information asymmetries.
- It is critical to ensure people’s awareness of their rights to provide feedback, and of available and easily accessible feedback channels, within a non-threatening environment in which people can freely express their views.
- Clear policies and operating guidelines with staff support and dedicated resources, will enable health workers to value, document and analyse information from patient feedback; and communicating timely actions taken helps maintaining people’s satisfaction with, and trust in, their health systems.

## Background

Effective interactions between patients and health systems are critical to well-performing systems.^1–4^ Well-functioning patient feedback systems help improve healthcare quality and ultimately accountability and responsiveness of national health systems.^2 3 5–9^

Effective feedback systems involve two key features. First is the supportive environment for patients to provide feedback on their experiences.^1 5 10^ Second is the health system’s ability to adequately respond to, and act on feedback.^1 6 8^ Substantial research covers patient feedback systems, their typologies, assessments and contributions to service quality improvement.^1 6 9 11–15^ However, most evidence comes from high-income countries and hospital settings, with less research covering lower-income countries and frontline health facilities.

We report a realist evaluation of patient feedback systems at local-level health facilities in Bangladesh. Our key question is: what about the patient feedback systems has worked, for whom, in which circumstances and why? Our findings should be of interest to academics and practitioners interested in understanding and improving, patient feedback systems and wider health systems performance.

### Theoretical underpinnings

Two theoretical perspectives underpin patient feedback systems. First, providing feedback is an act of citizenship involving social identity and practices by people with different capacities and resources within political and social structures and institutions.^16–19^ As Lister explains^20^ (p41) *‘*To be a citizen in the legal and sociological sense means to enjoy the rights of citizenship necessary for agency and social and political participation. To act as a citizen involves fulfilling the potential of that status’. Thus, citizenship is both an identity and a practice.^16 17 21^ Understanding of identity shapes one’s exercise of citizenship within social spaces (home, community, institutions, national politics, the global arena). People have different capacities and resources to express their citizenship identity within contexts of sociopolitical opportunities through available places and spaces.^16 18 19^ Expressions of citizenship include political forms such as voting in elections^16 21–23^ and patient feedback systems provide platforms for people’s engagements in decision-making within health facilities.

Second, multiple relationships between healthcare providers, patients and managers, occur within contexts of entrenched bureaucratic and professional hierarchical roles and relational dynamics.^24–27^ The Principal–Agent (PA) theory therefore helps understand these relationships. It postulates that all organisations require employer–employee cooperation.^24^ Using the metaphor of a contract, the PA theory highlights the agency in the relationship where the principal delegates work to the agents,^25 26^ for example, health managers delegating work to health workers, respectively. Central to this are the goal conflicts and differing preferences within hierarchical relationships. Two assumptions contribute to potential agency loss, a common metric for determining whether agents act in the principal’s interests^26^: diverging and independent interests of each party and an information asymmetry with agents being generally more knowledgeable of the local circumstances including their efforts and capacities. The PA theory seeks the most efficient contract with assumptions about the individuals (eg, self-interest, bounded rationality, risk preferences), the organisations (eg, goal conflicts) and information (ie, acquired commodity leading to information). Patient feedback systems involve relationships between three groups (patients, healthcare providers and managers) within contexts of information asymmetry and potentially diverging expectations.

### Context

Bangladesh comprises 8 divisions, 64 districts, 481 subdistricts (Upazilas) and 4403 unions. Most health budget is earmarked for Upazila Health Complexes (UHCs), making them a backbone of the country’s public health system. UHCs serve a population of 200 000–400 000, offer both inpatient (31–50 beds) and outpatient services, and act as first level referral for community clinics and village (union) health centres. UHC has between 93 and 128 staff, including 9–20 doctors, 13–16 nurses, 2 pharmacists and 2–5 laboratory technicians.^28^ UHCs provide preventive and basic curative services, have an ambulance and a pharmacy. A health management committee comprising local politicians, facility managers, civil society representatives and local leaders, monitors UHC work.

Improving health system’s accountability to the population is high on the policy agenda.^29^ An overarching framework, known as a Citizens Charter, summarises patient rights within public health facilities such as the right to receive affordable healthcare and with appropriate dignity and respect. This Charter is typically displayed at the entrance to public health facilities (online supplemental file 1).

10.1136/bmjgh-2020-004357.supp1 Supplementary data

Multiple centrally and locally managed patient feedback systems operate at UHCs (table 1). Their common strength is the underlying political commitment to enabling citizens voice and accountability, whereas common weaknesses include unclear processes and limited promotion of available channels. Unlike the locally managed, the centrally managed systems tend to have more functional record-keeping.

**Table 1:** Patient feedback systems in public health facilities in Bangladesh (data from informal stakeholder engagements)

System	Frequency	Feedback flow	Key strengths	Key limitations
Online grievance redress system	Unknown	Post→database→cabinet→implementer assigned→resolution→report back complaint	Anonymous, if registered get SMS updates. Instructions, contacts explained. Focal person from each ministry. Online breakdown of cases by ministries.	A system not seemingly functional. Public awareness is limited. No monitoring with local level. Accountability chains unclear.
SMS texting system	About 95 monthly for country	SMS→MOHFW verifies→call UHCs→priority assigned, solution→steps logged	Texts anonymous. Instructions on boards at facility entrance. Verification of cases, local resolution. Online data available by type, time, facility.	No promotion, other than boards at UHCs. Inaccessible to illiterate, with no mobiles. No guidelines or policies. Processing, follow-up by one person only.
Call centre—16 263	Over 15 daily	Call→operator registers feedback, name, address→MIS→investigation, resolution→report back	Patients can feedback anonymously. Toll-free number promoted by flyers and posters at health centres. Online breakdown by types and time.	Promotion at the sub-district not evident. No systematic documentation. Feedback processing and follow-up unclear to the public.
Verbal	Unrecorded reportedly frequent	Contact staff→feedback→resolution	Verbal feedback to UHC head, emergency department, staff. Immediate response.	No guidelines about lodging. No records kept.
Written letters	Unrecorded reportedly very rare	Letter to management committee→investigation→resolution→report back	UHFPO assigns focal person, monitors. Committee decisions published online.	Unused, broken, boxes, no instructions. No written record kept. No assigned person for the feedback.
Complaint box	Rarely used	Written feedback→report the head of UHC→resolution, reporting	Anonymity possible. Local resolution at UHC. Convertible to digital format by scanning.	No assigned person available. Boxes often broken or closed.

MoHFW, Ministry of Health and Family Welfare; UHC, Upazila Health Complex; UHFPO, Upazila Health and Family Planning Officer.

## Methods

Given our interest in understanding ‘what about the patient feedback systems has worked, for whom, in which circumstances and why’, we chose to conduct a mixed-methods realist evaluation. A realist approach was deemed appropriate because it entails a theory informed critical examination of a programme’s logic within its context, articulated as a programme theory (PT).^30^ PTs represent hypotheses to be subsequently tested, refined and consolidated. Researchers interrogate their initial PTs through identifying causal pathways of how specific mechanisms (reasoning and resources) are triggered in different contexts, to produce (un)intended outcomes. These pathways are articulated as Context-Mechanism-Outcome configurations^31–33^ (CMOs). The refined PT is based on the evaluation about what aspects of the intervention worked, for whom, in which conditions and why. RAMESES II standards for reporting realist evaluations^34^ guided this paper.

The study was conducted in two UHCs of Comilla district which neighbours the capital Dhaka, and has one of the highest feedback rates. This selection was based on: (a) analysis of publicly available web portal of patient feedback data and (b) non-participant observations of patient feedback environments.^35^

**Figure 1:**
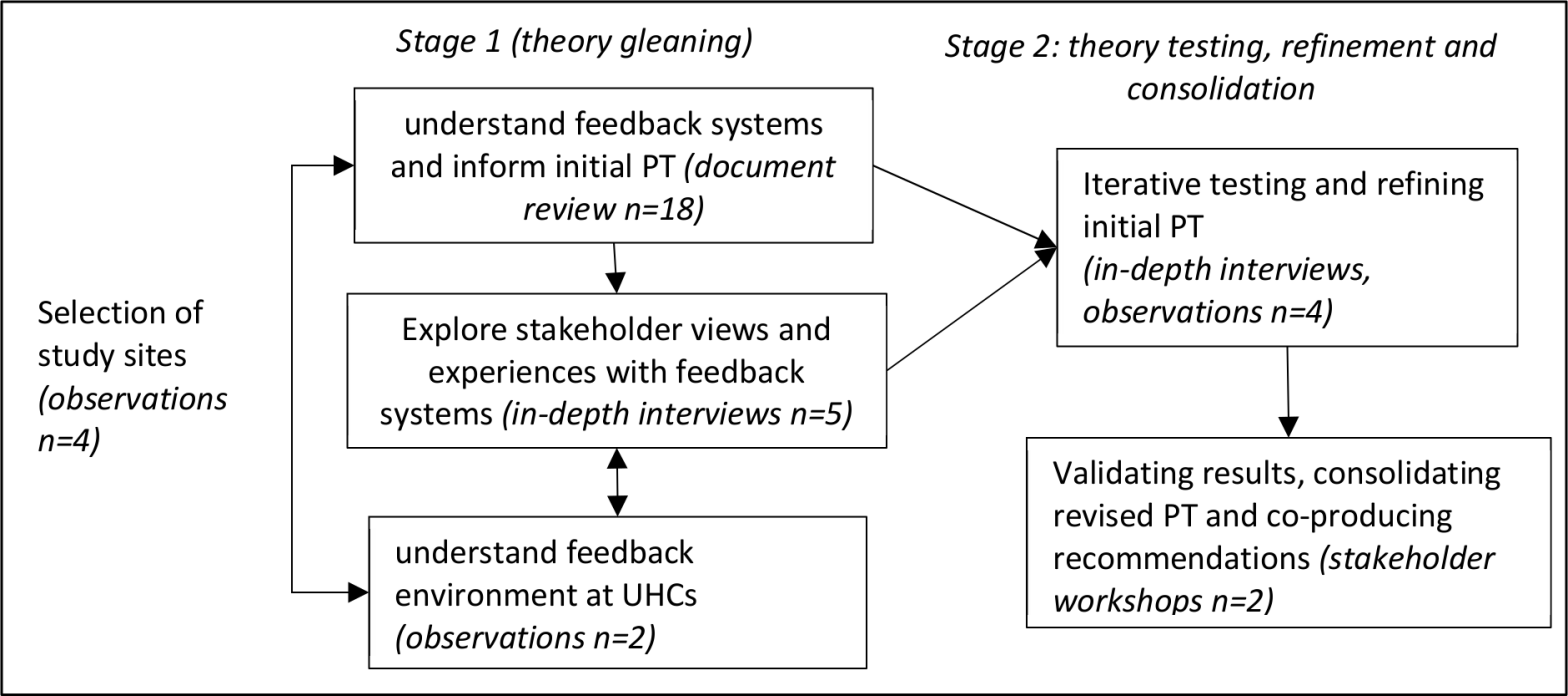
Study methods across the two stages. PT, programme theory; UHCs, Upazila Health Complexes.

We collected data in two stages, using different methods (figure 1).

Stage 1 was exploratory and focused on identifying the initial PT, using the literature, perspectives of policymakers, facility managers and patients, to understand the logics behind patient feedback systems. We began with a review of design-related documents: practice guidelines, which articulated processes of the feedback system and the different roles involved; job descriptions of involved personnel; internal reports and news items about patient grievances. In total, 18 documents were reviewed; these were obtained from Directorate General of Health Services, Ministry of Health and Family Welfare (MoHFW) and through a web search.

We then conducted in-depth interviews (n=5) with a small number of purposefully selected public representative of the Upazila council, two national policymakers and two health facility managers, to understand their perspectives. All interviews were transcribed verbatim and uploaded into NVivo for coding by the contexts, mechanisms and outcomes. We conducted a stakeholder workshop to glean their understanding of how the feedback system was supposed to work, and elaborate specific CMOs. Throughout stage 1, we explored substantive theories which could help frame the logics underpinning patient feedback systems and the specific CMOs.

Non-participant observations informed the selection of study sites and were also conducted during stage 1. The rationale was to complement resource-intensive interviews, and gaps in feedback documentation (table 1). Observations lasted between 30 min and 2 hours and covered degree of visibility, user-friendliness, utilisation and maintenance of key feedback channels in the UHCs such as suggestion boxes or telephone hotlines. A semistructured observation checklist (online supplemental file 2) UHCs was used.

10.1136/bmjgh-2020-004357.supp2 Supplementary data

In stage 2, we iteratively tested and refined initial PT and its detailed CMOs, and at the end of this stage we eventually consolidated the revised PT. At this stage, we used data from observations and in-depth interviews (n=20) with 10 health staff and 10 patients from UHCs. The initial PT provided the basis for the interview guide and for the checklist for observations. The interviews were also oriented towards interrogating the veracity of aspects of the initial PT. Each interview lasted 25–60 min, was audio-recorded and transcribed verbatim, translated into English where required and uploaded into NVivo where coding was now more driven by elements of causality within the CMOs. Two researchers conducted non-participant observations, using a checklist of presence of feedback materials and processes, and functioning of the feedback system.

At this stage, we conducted two stakeholder workshops. These lasted 4–5 hours and each involved 20–25 representatives from government (12–15), non-government (4–5) and international organisations (4–5). Workshops included presentations of emerging findings to inform a plenary discussion, and then more in-depth work in smaller groups to validate results. While the primary aim of these workshops was to share and validate emerging results with key stakeholders, proceedings were audio-recorded following informed consent and were treated as further data for analysis.

A retroductive approach to data analysis^36 37^ was used throughout the study. It combined both inductive and deductive logics to identify often hidden generative causation within our PTs. This included iterative engagements with: (a) data from interviews, documents and observations, which were analysed by local researchers in Bangladesh who were trained in realist evaluation and then extensively discussed among the team; (b) the underpinning theoretical and relevant empirical literature which was continuously identified and reviewed and (c) engagements with key stakeholders throughout the study and during two workshops.

## Results

**Figure 2:**
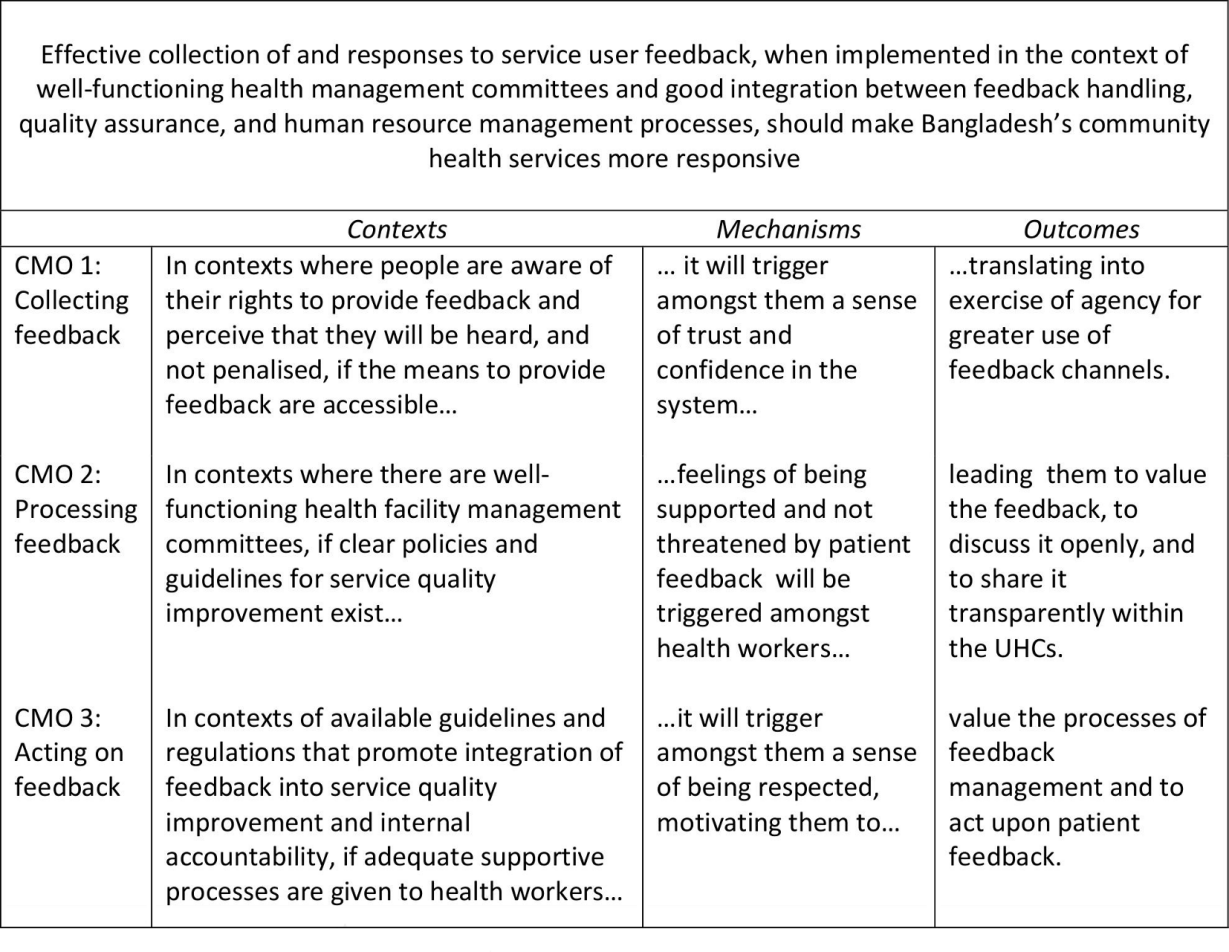
Initial programme theory. CMO, Context-Mechanism-Outcome; UHCs, Upazila Health Complexes.

Our initial PT (figure 2) was gleaned from iterative document review, stakeholder perspectives from initial interviews and literature.

Our initial PT comprised three CMOs, which reflected three steps in patient feedback management.^15^ As we elaborate next, during stage 2 of the study these CMOs were iteratively tested and validated against the data and the literature.

### Collecting feedback

The interviews revealed that patients were generally aware of their rights to express opinions. This reflected the context of Bangladesh with vibrant grassroots-level citizenship. However, as our observations also revealed, many patients did not know about available feedback channels:

In this health complex … [laughing with a bit of incredulity], I am not aware of … such a thing. To my knowledge, there isn’t any feedback system … [with confusion]’ (Patient: 005)

Most managers concurred and shared the view that while feedback systems existed at UHCs, patient awareness of both their rights to and of available feedback channels, was limited. Service providers reflected that lack of awareness was due to a mix of low literacy and insufficient system’s efforts:

Basic reason for low awareness is poor education. Another reason is information gap … they do not know about the systems. (Provider: 003)

Many available feedback channels were not accessible. Observations revealed that the information about the SMS system was on a whiteboard on a wall, but at a not easily visible height (online supplemental file 1). While suggestion boxes existed at every UHC, most were not clearly labelled. Consequently, most remained either unused or even misinterpreted as donation boxes which tend to be ubiquitous in public locations in Bangladesh (online supplemental file 1). As a result, many felt what one patient said ‘The [SMS] board and the [suggestion] box failed to attract my attention. They are not eye-catchy’ (Patient, UHC).

Social access to feedback channels was often more important than physical. In Bangladesh, the socioeconomically disadvantaged often perceive themselves as not deserving of raising voice against the unfairness they encounter, as illustrated in the following quotes.

A patient like me is not likely to discuss such issue … If I say something, they wouldn’t listen. It would prove helpful, but I do not think I can have the capacity … (Patient: 006)

People like you and me … we know that we can protest against anything wrong … However, those who come from villages … they simply accept the mistreatments in silence. They do not even know how to complain or whom to complain to. If someone asks for a bribe, they simply bribe the person to get help. (Patient: 007)

In the second excerpt a socioeconomically well-off patient explains these class differences. This poverty of agency, and the lack of access among those at the bottom of the socioeconomic hierarchy is well recognised in Bangladesh. However, the multiple feedback channels do not sufficiently recognise this reality and fall short in supporting feedback from most vulnerable.

Our analysis also revealed low levels of trust in the feedback processes at UHCs and the wider health system. Patients’ distrust and their resultant hesitation to provide feedback, were rooted in their doubts about the benevolence of the health system and in fear of consequences:

Because when we try to say something, we are afraid of not getting treated properly or be harassed. There is always a fear and we do not say anything. As it happened to me, when I complained about something, they asked me to submit a written complaint. I did not agree because of fear … what if I was harassed afterwards? However, if there was a system where I could complain, but by hiding my identity, it would be better. (Patient: 010)

Fears of retribution and distrust of the system’s ability to act fairly, consistently featured as logics underpinning people’s decisions to not provide feedback. While some expressed this openly, others were more reticent. Many providers recognised this, but proffered rather simplistic explanations effectively dismissing these fears as unfounded.

They have no reason to fear. It is due to ignorance and lack of education. Sometimes, when the patients make verbal complaints, we advise them to place written complaints. However, they do not agree. (Provider: 003)

Such explanations reflect a disconnect between providers and patients. They spotlight entrenched prejudices and the class divide whereby the privileged inhabit public systems and view the under-privileged as being ignorant. Such social relational dynamics are fundamental to people’s distrust of the health system, manifested in multiple calls from patients for anonymisation. The non-anonymised feedback systems are therefore under-used. Consequently, people provide feedback through their acquaintances.

Most of the time, they express their opinion to those persons who are very familiar to them. Suppose a sweeper or cleaner … (Provider: 004)

As the quote illustrates, personal connections were preferred feedback routes. While this reflects low levels of trust in the system, it could also reflect preferences for relational ways of interacting in the society, rather than processual bureaucratic ways that current feedback channels offer. When queried about a possibility of low trust being the reason for the limited uptake of feedback channels, providers pointed to the high use of services as a counterargument. While this was plausible, it is more likely that the high service use merely reflects lack of alternative healthcare options.

Our findings are consistent with the literature which highlights awareness about rights as a prerequisite to exercising one’s agency.^38–42^ Our findings highlight that while being aware is necessary, mere awareness is not enough for exercising agency and rights, echoing the literature on limited feedback by the socioeconomically disadvantaged in Nepal, Russia and Israel^11 41 42^ and high-income contexts such as the UK.^43^ Fears of retribution and distrust of the system consistently underpin people’s decisions not to complain.^11 41 42 44–46^ Patients distrust feedback systems because they doubt the benevolence of the health system and are afraid of retribution, which undermines their agency as the likely principals when providing feedback. Conversely, if people were to perceive that they will be heard and not penalised for their views, they are likely to trust the system and use available channels. This supports our initial CMO which posits that awareness of rights combined with accessible channels, will trigger a sense of translating into exercise of agency.

Preferences of informal feedback have been widely reported across public services.^7 47–49^ This suggests that giving feedback involves culturally mediated processes governed by local social norms about acceptable conduct. This is particularly so where formal processes are weak or trust in the system is low, or where there is general preference for relational ways of interacting.

### Processing feedback

The centrality of clear policies, guidelines, processes and roles and fostering overall system’s accountability and transparency, emerged as a key finding. One provider emphasised that ‘Of course, a guideline is needed. Without a guideline, the process cannot be maintained in an organised way’. This limited clarity was a critical missing link that led to different interpretations of and practices around feedback management. It also seemed to underpin the apparent lack of shared goals between policymakers, local managers and providers:

I think it is also important to know who is designated for which work. It is necessary to know who has what authority. There needs to be a guideline. For instance, detailing where to begin and whom to go to; what is the process to arrive at a resolution (Provider: 010)

These views were echoed by patients, particularly those with good knowledge of the healthcare system:

… I would want to know: who is in charge, who will work on it, how will they, then who is going to solve it? If it is solved, how will they inform the patients? Every step should have specific guidelines (Patient: 010)

Clarity about roles and responsibilities could help bridge the information asymmetry gap between the patients and the healthcare providers, ultimately enabling people to exercise their agency in providing feedback. Multiple providers consistently viewed this dysfunction as a management failure. They pointed to an ad-hoc nature of feedback management, and how ‘Complaints have never been taken by us positively’; expressing displeasure ‘If a process existed, then our good officers would not suffer like this’ and articulating the desire for fair and non-punitive feedback systems *‘*If there were guidelines … regarding complaints, and if it were clearly written, I think that would bring some transparency to the process …’.

These quotes highlight the cooperation challenges between the Upazila Health and Family Planning Officers (UHFPOs) and health workers. The UHFPOs do not always know the details of strengths and limitations of efforts by providers. UHFPOs, perhaps understandably, use patient feedback as a lever to extract accountability from staff. The absence of transparent documentation of feedback means that such tactics by UHFPOs attract resentment from staff. In the context of weak feedback management processes, the UHFPO’s tactics can undermine the learning potential from patient feedback and fuel staff resentment towards feedback systems.

A key constraint to feedback processing was the unavailability of dedicated staff with relevant expertise. Observations revealed that UHCs were generally understaffed and feedback management was an additional responsibility for clinical staff. Managers and health workers recognised these constraints. Providers argued that ‘An extra person be recruited and given the responsibility to maintain [feedback system]’, adding that

Because when a doctor is in an emergency and there are five patients waiting for him, if anyone calls the [complaints phone] number, documenting the feedback from that call at that time would be very difficult. That is why dedicated human resources are needed (Provider: 003)

Document review revealed that the UHFPO, a medical doctor, is responsible for all administrative issues. All UHC staff are answerable to the UHFPO who reports upwards to the Director General (DG) at MOHFW. However, the UHC as a public service is also accountable to the Upazila Chairperson, Upazila Nirbahi Officer, Member of Parliament, religious leaders and local community members—all members of the UHC Management Committee. This committee, however, is chaired by the UHFPO, who is then responsible for communicating all issues upwards to higher health authorities.

One provider summarised the UHFPO as: ‘Actually here everything happens under the command of UHFPO. Ok! He can do these activities here. Everything is controlled by him’ (Provider: 003). Much power and authority lies with the UHFPO to the extent that across the two UHCs the management committees were non-functional, suggesting that accountability runs primarily vertically, and only notionally horizontally.

I am a member of some committee, but the fact is, I do not know because the position was given to me in the Upazila coordination committee’s meeting … I have been Chairman for about eight months. That [attending meetings] never happened. (Local leader: 001)

In line with the aforementioned, key actors did not deem it worth their while to attend these meetings. The following quote highlights usual practices that UHC in reality is de-facto answerable to the DG, MOHFW.

We have a management committee; the Parliament Member is the president of this committee. Other members are the Upazila Executive Officer, Upazila Chairman, Union Parishad Chairman, and few community people. All members are supposed to attend the monthly meetings. However, in reality, only we, the doctors of this facility remain present … [and] upload the meeting minutes in the MIS [management information] system of DG office. (Provider: 001)

Many providers related this disengagement to absence of mechanisms for members to exercise their authority in the committee. However, this contradicts the fact that most members outrank, and have no need to favour, the UHFPO. The most plausible explanation is that local leaders do not see sufficient political value in these committees and health is a generally low political priority in Bangladesh.

The literature highlights that clear quality improvement guidelines and supportive policies facilitate well-functioning patient feedback systems.^45^ Similar to our results, studies have shown that frontline workers need support to manage feedback effectively and that adequate supervision can help staff value patient feedback^6 44 50 51^ and consequently align somewhat divergent objectives of two principals: patients and UHFPOs. However, the mere existence of guidelines is not enough, frontline workers need to be aware of feedback processes and require skills to deal with often difficult interactions.^50^ Together with our findings, this supports our initial CMO that highlighted clear policies and guidelines as being important in triggering health workers’ feelings of being supported, not threatened and valuing patient feedback.

However, we found that a critical contextual aspect of our initial CMO was missing. The UHC management committees were non-functional with non-involvement of local leaders, which undermined their roles as principals with regards to the UHFPOs. Substantial literature has examined the conditions under which local social accountability structures can fulfil their mandates.^52–55^ It spotlights that constructive and sustained local political involvement is a key to active local accountability structures, and improvements in service quality, responsiveness and equity. This counterfactual analytical rendering of what was amiss in our study context thus supports the logic of our initial CMO.

### Acting on feedback

Robust regulatory framework and institutional support were seen by all interviewees as being crucial to enable staff to act on feedback. This view was echoed in the stakeholder workshops and in national-level documents reviewed in the study. Participants agreed that training of providers, clear Terms of References or guidelines, explicit roles, and resources were critical to effective feedback management. While there were plans to introduce such frameworks by the MOHFW, none were yet in place.

During interviews, most providers revisited their initial resentment towards feedback systems. Many reflected that ‘Through the feedback, at least our work would get (some) appreciation’, recognised its value in being able to ‘praise the good performance and to punish misdeeds’, and appreciated the learning opportunities from feedback, saying that ‘[*in response to feedback*] exemplary action should be taken so that with one example, others become cautious’. Many added that any effective regulatory framework should include an appropriate balance between incentives and sanctions, arguing for links with staff appraisal, rewards and recognition.

Patients accorded high importance to the user-friendliness of feedback channels and that actions on feedback ought to be transparently communicated to enhance the credibility of the feedback.

It is better to inform patients because in this way they will get to understand that through this system they solved my problem. This patient will spread it to others. (Patient: 005)

This centrality of open, transparent communication between service providers and users to creating trust and improving staff–patient relationships was also consistently recognised by providers.

… if we can inform the patients about the solution, they will be pleased thinking that their complaints led to some solutions. By being happy, they will encourage their neighbours, thinking that problems are being solved and communicated well. It will improve the relationship between hospital and patients. (Provider: 001)

The literature echoes our findings and adds that while policies and guidelines can catalyse action on feedback, unsupportive institutional cultures and ineffective communication skills of service providers may hinder the desired effects.^8 43 56^ Furthermore, a receptive and learning institutional culture can help staff recognise the value of transparent and fair feedback management.^43 57^ Consistent with our results, scholars have found that transparency in feedback management can enhance the health system’s credibility and foster patient trust,^58^ thus contributing to bridging the information asymmetry and alleviating potential losses of agency. This confirms our initial CMO which linked clear guidelines and processes along with support to facility staff, with a sense of being respected and motivation to value and act on feedback.

## Discussion

**Figure 3:**
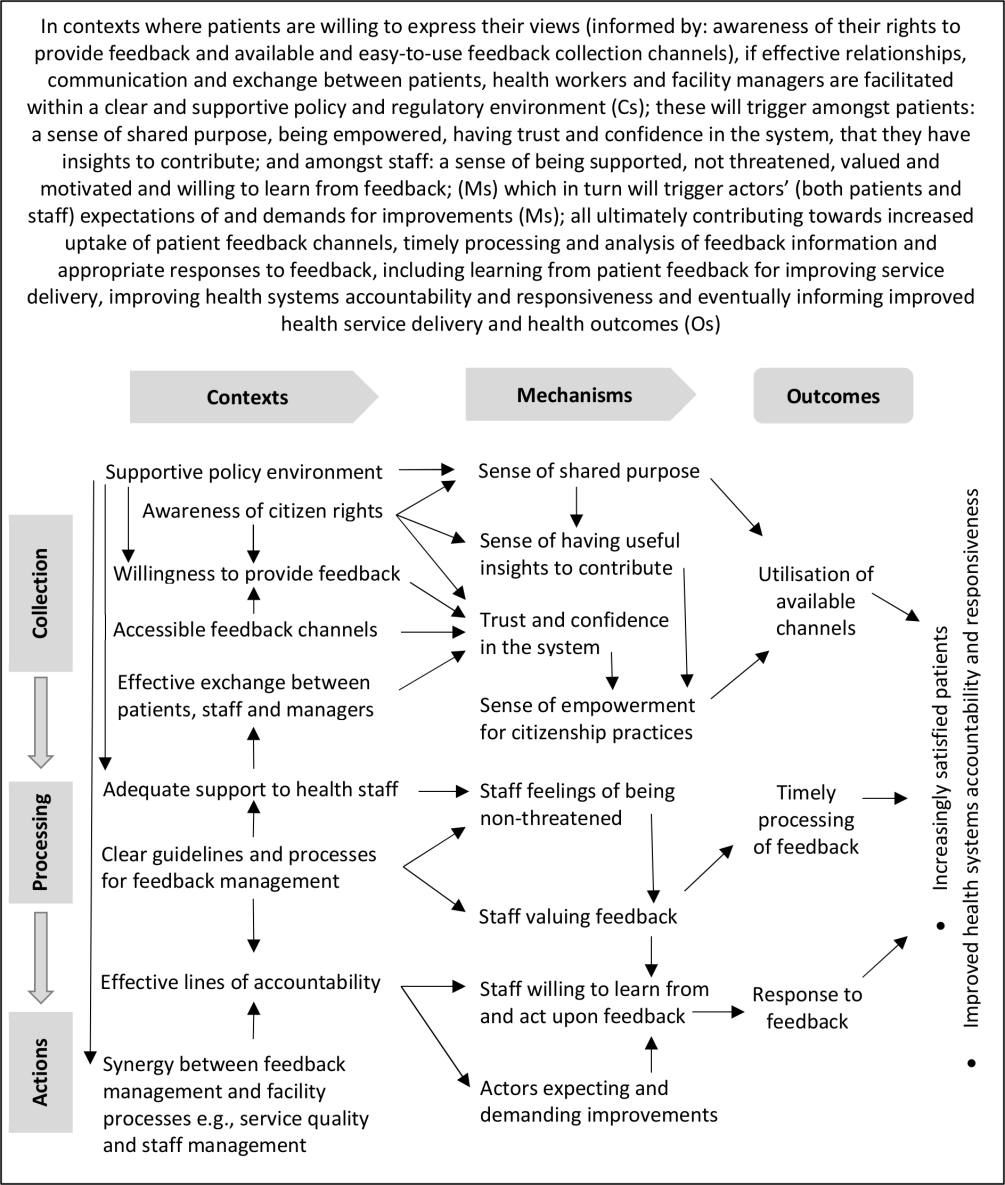
Refined programme theory of patient feedback systems in Bangladesh.

Our revised PT (figure 3) was consolidated following testing and refining throughout data collection and analysis and against the literature.

The testing of CMOs revealed the contingent nature of triggering of mechanisms, and variable achievement of outcomes. Our revised PT highlights this contingency, serving three objectives: first, it helps show the many ways in which feedback systems (not) operate to (not) achieve their intended outcomes.^30^ For example, our findings highlight the importance of awareness about one’s rights for patient feedback, but the actual provision of feedback can be constrained by socioeconomic disadvantage and fears of retribution. Second, it speaks to a central tenet of critical and scientific realism—of causality.^59^ Further to numerous CMO pathways illustrated through arrows in figure 3, our results also reveal causality among specific contexts (eg, supportive policy and management environment contributes to effective exchange between patients and staff) and mechanisms (eg, patients’ sense of empowerment and staff’s sense of being supported contribute to actors’ expectations of improvements) and arguably contribute to advancing theorisation around causality within complex programmes. Third, the ‘if-then’ propositions reflected in the narrative of figure 3 and multiple causal pathways, can serve as a practical heuristic for informing future interventions and policies, as argued elsewhere.^60 61^ The contingent nature of contexts, interventions, mechanisms and outcomes also further highlights the inter-related three steps of the feedback process.

Awareness of feedback channels and people’s trust in health systems are important determinants of people’s citizenship identity and willingness to exercise their agency. Awareness and trust are, however, insufficient and need to be bolstered by recognition of rights to provide feedback, accessibility of feedback channels, clear policies and guidelines, and appropriate incentives and sanctions to ensure staff compliance.^1 5 15^ In line with published evidence,^1 5 8 62^ a key determinant of decisions to provide feedback is people’s confidence not to be disadvantaged after providing feedback. Societal preferences for relational ways of interacting and social norms about appropriate ways of expressing dissatisfaction also shape the use of feedback channels.^58^

The primacy of vertical accountability in UHCs raises many questions. The UHFPO’s leadership has its advantages. The UHFPOs are the principals vis-à-vis the health workers but are the agents vis-à-vis the DG and the patients. Effective performance of being a principal and an agent role by UHFPOs is likely to be difficult and untenable. The UHFPOs’ current role reinforces hierarchies and concentrates power. This may demotivate staff from learning from feedback within health facilities^1 43 62^ and may prevent some patients from providing feedback and instead exerting violence.^63 64^ Weak horizontal accountability, evidenced by non-functional management committees and lack of engagement from local leaders, may reflect political realities, and that people have to turn to own social networks to redress grievances, is problematic. From an equity perspective, reliance on social networks to express grievances systematically disadvantages the exercise of citizenship by those with the least social and relational capital. Weak horizontal accountability is a missed opportunity, given that literature attests to potential quality, equity and responsiveness gains through local accountability processes.^52–55^ While in the immediate future, improvements to patient feedback systems could leverage the currently dominant vertical accountability, it would be critical to recognise its limitations in providing equal opportunities and spaces for those most disadvantaged.^18 19^ This literature notwithstanding, our findings caution against a universalist normative understanding that healthcare can be held to account through local political structures in all contexts. Our findings highlight that where health is not a political priority and where local leaders are not answerable to people, horizontal arrangements like the management committees at UHCs, are unlikely to be equitably effective.

We explored the application of PA theory in healthcare settings. Our findings suggest that the two fundamental tenets of PA theory (information asymmetry and divergent goals) are less clear-cut within patient feedback systems. Managers, providers and patients can be both principals and agents. Such blurred identity and relational boundaries highlight the multiple, dynamic and often conflicting, roles and responsibilities within principal–agent relationships^27^ at the frontline of healthcare provision. Our results also extend the understanding of PA theory in two inter-related ways. Patient feedback systems can loosen information asymmetry between the agents and principals, for example, through patients communicating information about the health workers’ conduct to the managers. Consequently, feedback systems can therefore contribute to alleviating agency losses, for example, through health workers empowering patients by sharing actions taken in response to their feedback.

Combining the citizenship and PA theories has allowed us to gain insights into the logics underpinning three steps of patient feedback processes.^15^ People’s use of available feedback channels entails people expressing their citizenship and agency, within the context of interpretations of one’s identities and power relations and information asymmetries between patients and healthcare providers.^16 18 19 26^ Adequate processing and analysis of patient feedback is contingent on health workers’ willingness to engage with feedback within the context of in-situ organisational dynamics, target-setting and staff performance management.^6 44 50 51^ Actions on patient feedback, including reporting back to patients, entail bridging of information asymmetries across various principal–agent relationships, and enabling the expression of citizenship and exercise of agency of patients, health workers and facility managers alike.^20 26 57^

We propose three implications for future health policy and practice. First, health systems should ensure and maintain people’s awareness of their rights to provide feedback, and of available and easily accessible feedback channels, within a non-threatening environment in which patients can express their views without fears of subsequent retribution. Second, clear policies and operating guidelines with adequate support and dedicated resources, will enable health facility workers to value, document and analyse information from patient feedback. Last but not least, communicating timely actions taken in response to the feedback will help maintain people’s satisfaction with, and trust in, their health systems and will help maintain the rapport between the people, health workers and managers.

### Study limitations

We recognise two study limitations. First, our inquiry was framed within two substantive theories, and while we feel using citizenship and PA theories has allowed us to understand the logics of patient feedback systems and advance the understanding of these theories, future studies can anchor their inquiries in other theories such as on relational trust or motivation. While we consider our refined PT being comprehensive given our robust study design, grounding inquiry in other substantive theories may enhance or even add further dimensions to the CMO configurations. Second, we examined established patient feedback systems at selected grassroots-level health facilities and in one district and country only, and future research can test our refined theory in hospital settings, further countries and possibly feedback systems which involve substantial informal processes and engagements.

## Conclusions

Appropriate policy frameworks and clear implementation processes and explicit consideration of historical, social and institutional relational arrangements, are key to the design and effective implementation of complex programmes such as patient feedback systems. Further, in contexts where there is a preference for relational ways of interaction, people will exercise their citizenship and agency to provide feedback only if they can trust the health system.
