# Supplementary material for: How do patient feedback systems work in low-income and middle-income countries? Insights from a realist evaluation in Bangladesh
Source: BMJ Glob Health. 2021 Feb 10;6(2):e004357. doi: 10.1136/bmjgh-2020-004357 (PMC7878124; doi:10.1136/bmjgh-2020-004357)

Supplementary file 1

Photo of the Citizens Charter

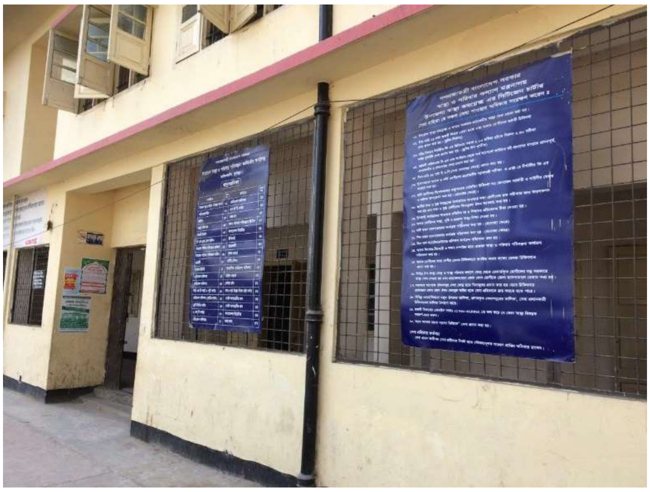

Photo of an SMS board at health facility

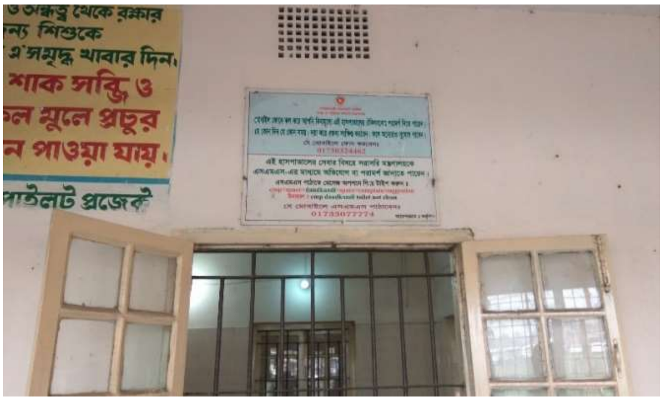

Photo of a Suggestion Box at a health facility

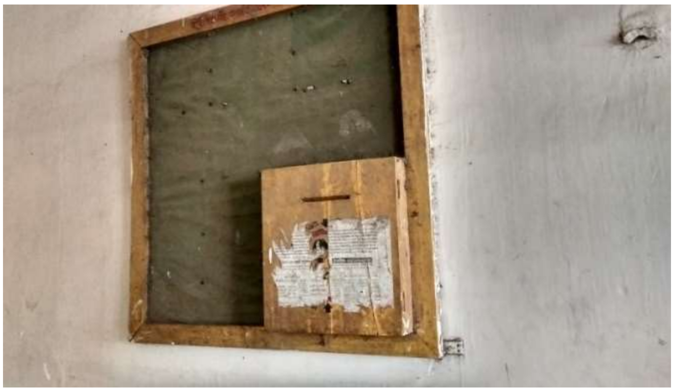

Supplement: Supplementary data [file bmjgh-2020-004357supp001.pdf]
