# Supplementary material for: How do patient feedback systems work in low-income and middle-income countries? Insights from a realist evaluation in Bangladesh
Source: BMJ Glob Health. 2021 Feb 10;6(2):e004357. doi: 10.1136/bmjgh-2020-004357 (PMC7878124; doi:10.1136/bmjgh-2020-004357)
Supplement: Supplementary data [file bmjgh-2020-004357supp002.pdf]

# Creating responsive health systems: improving the use of feedback from service users in quality assurance and human resource management in Bangladesh (RESPOND project)

## Observation proforma

*Instructions:*

- *Please add your observation notes in the second column, with any examples where possible*
- *Please use this form flexibly and add further rows as appropriate.*

Location / facility name ___________________

Researcher completed ________________ Date ______________________

| Item | Observation notes |
|---|---|
| Which feedback system(s) are available in health facility? | |
| How visible are these systems in health facility/ward? | |
| How easy to use or user-friendly these are? | |
| How well these are being utilised? | |
| How well these were maintained in health facility? | |
| Any other comments? | |
